# The impact of inflammatory factors on patients with concurrent lung cancer and acute pulmonary embolism

**DOI:** 10.3389/fcvm.2025.1636717

**Published:** 2025-10-15

**Authors:** Meizhi Li, Qiong Yi, Shangjie Wu, Yuan Li, Fang Li

**Affiliations:** ^1^ Department of Pulmonary and Critical Care Medicine, The Second Xiangya Hospital of Central South University, Changsha, China; ^2^ Department of Medical Administration, The Second Xiangya Hospital of Central South University, Changsha, China; ^3^ Research Unit of Respiratory Disease, Central South University, Changsha, Hunan, China; ^4^ Clinical Medical Research Center for Pulmonary and Critical Care Medicine in Hunan Province, Changsha, China; ^5^ Diagnosis and Treatment Center of Respiratory Disease, Central South University, Changsha, China; ^6^ Hunan Research Centre for Evidence-based Medicine, The Second Xiangya Hospital of Central South University, Changsha, China

**Keywords:** lung cancer, acute pulmonary embolism, hyperuricemia, thromboinflammation, neutrophil-to-lymphocyte ratio, platelet-to-lymphocyte ratio

## Abstract

**Introduction:**

Lung cancer is a leading cause of cancer-related mortality globally, with acute pulmonary embolism (APE) significantly worsening the prognosis of affected patients. Inflammatory pathways are increasingly recognized for their dual role in both oncogenesis and thrombotic events. This study aimed to investigate the prognostic significance of specific inflammatory biomarkers, particularly neutrophil-mediated mechanisms, in lung cancer patients complicated by APE.

**Methods:**

A retrospective cohort analysis was conducted on 90 lung cancer patients admitted to the Second Xiangya Hospital of Central South University between January 2019 and December 2022. Propensity score matching (PSM) was employed to ensure balanced demographic and clinical covariates. Multivariate logistic regression analyses were performed to identify independent predictors of APE occurrence and disease severity.

**Results:**

Multivariate logistic regression identified elevated neutrophil count as an independent predictor of APE occurrence (adjusted OR = 3.068, 95% CI: 1.472–6.394, *p* = 0.003). In the subgroup analysis of APE patients, hyperuricemia (UA >260.1 μmol/L; OR = 1.017, 95% CI: 1.002–1.033, *p* = 0.028) and a reduced platelet-to-lymphocyte ratio (PLR < 318.83; OR = 0.990, 95% CI: 0.981–0.999, *p* = 0.037) were significantly associated with increased disease severity and ICU admission. Receiver operating characteristic (ROC) curve analysis validated the strong discriminative capacity of neutrophil count (AUC = 0.952), UA (AUC = 0.782), and PLR (AUC = 0.792) in stratifying APE risk and clinical outcomes.

**Discussion:**

Our findings highlight neutrophilia as a potential biomarker for APE susceptibility in lung cancer patients. Furthermore, elevated uric acid levels and a diminished PLR may serve as valuable indicators of disease severity in this high-risk population. The study underscores the critical need to integrate these inflammatory markers into standardized clinical risk assessment frameworks to optimize therapeutic strategies and improve patient management.

## Introduction

Patients with malignancies are known to exhibit an elevated risk of venous thromboembolism (VTE), clinically presenting as deep vein thrombosis (DVT) and pulmonary embolism (PE) (1). Among malignancies, lung cancer demonstrates a higher susceptibility to PE, with a pooled incidence of 3.7% (2, 3). Notably, 79% of PE cases originate from DVT, while the remaining 21% may arise independently, often linked to cancer-related thrombogenic mechanisms (4, 5). Acute PE (APE) can manifest suddenly and unpredictably, making it challenging to diagnose and leading to high mortality rates (6). The coexistence of APE and lung cancer can complicate the diagnosis and treatment of both conditions, and further elevate the risk of adverse events and mortality rates in affected individuals (7).

The early detection of lung cancer combined with APE can greatly enhance patient prognosis (8). To address these challenges, researchers have focused on developing innovative diagnostic tools and methods for early detection of APE and risk assessment (9). These include advanced imaging techniques such as computed tomography (CT) scans, positron emission tomography (PET) scans, and magnetic resonance imaging (MRI) (1, 10). Systemic inflammatory indices such as neutrophil-to-lymphocyte ratio (NLR), platelet-to-lymphocyte ratio (PLR), and lymphocyte-to-monocyte ratio (LMR) have emerged as potential prognostic biomarkers in acute PE (11). Studies have demonstrated that these markers may contribute to early risk stratification in patients with acute pulmonary thromboembolism (12, 13). Inflammatory markers, such as C-reactive protein (CRP), interleukin-6 (IL-6), and fibrinogen, have been widely studied in various diseases, including cancer and thrombotic disorders (14–16). These markers serve as indicators of both inflammation and poor prognoses. Although there is extensive research on the relationship between inflammation, thrombosis, and cancer, limited studies have focused specifically on the effect of inflammatory markers on lung cancer patients with concurrent thrombosis (17). Studying the impact of these inflammatory factors on lung cancer patients with APE could provide crucial insights into disease progression and potential therapeutic strategies (18). This study investigates the role of inflammatory factors in this high-risk cohort to elucidate disease progression and therapeutic targets.

## Methods

### Study design and participants

A retrospective analysis was conducted on medical records of lung cancer patients with or without VTE admitted to the Second Xiangya Hospital of Central South University between January 2019 and December 2022. Lung cancer patients were searched in the electronic medical record system according to the tenth revision of the International Classification of Diseases (ICD-10) codes. The diagnosis of lung cancer was pathologically confirmed. The diagnosis of APE was confirmed by computed tomography pulmonary angiography (CTPA) or pulmonary angiography and adhered to the 2019 European Society of Cardiology (ESC) guideline (19). APE was classified as symptomatic if clinical signs (e.g., acute dyspnea, chest pain, syncope) prompted diagnostic imaging, or incidental if discovered on imaging performed for other reasons (e.g., cancer staging). The diagnosis of DVT adhered to the National Institute for Health and Care Excellence (NICE) guideline (20). Control measurements were generated by analysing lung cancer patients with DVT and lung cancer patients without VTE by using Propensity Score Matching (PSM) based on sex and age. Ethical approval was obtained from the Second Xiangya Hospital of Central South University (2022-107).

The inclusion criteria comprised: 1) Confirmation of APE through computed tomography pulmonary angiography (CTPA) or pulmonary angiography; 2) hospitalisation time longer than 24 h.

**Figure 1:**
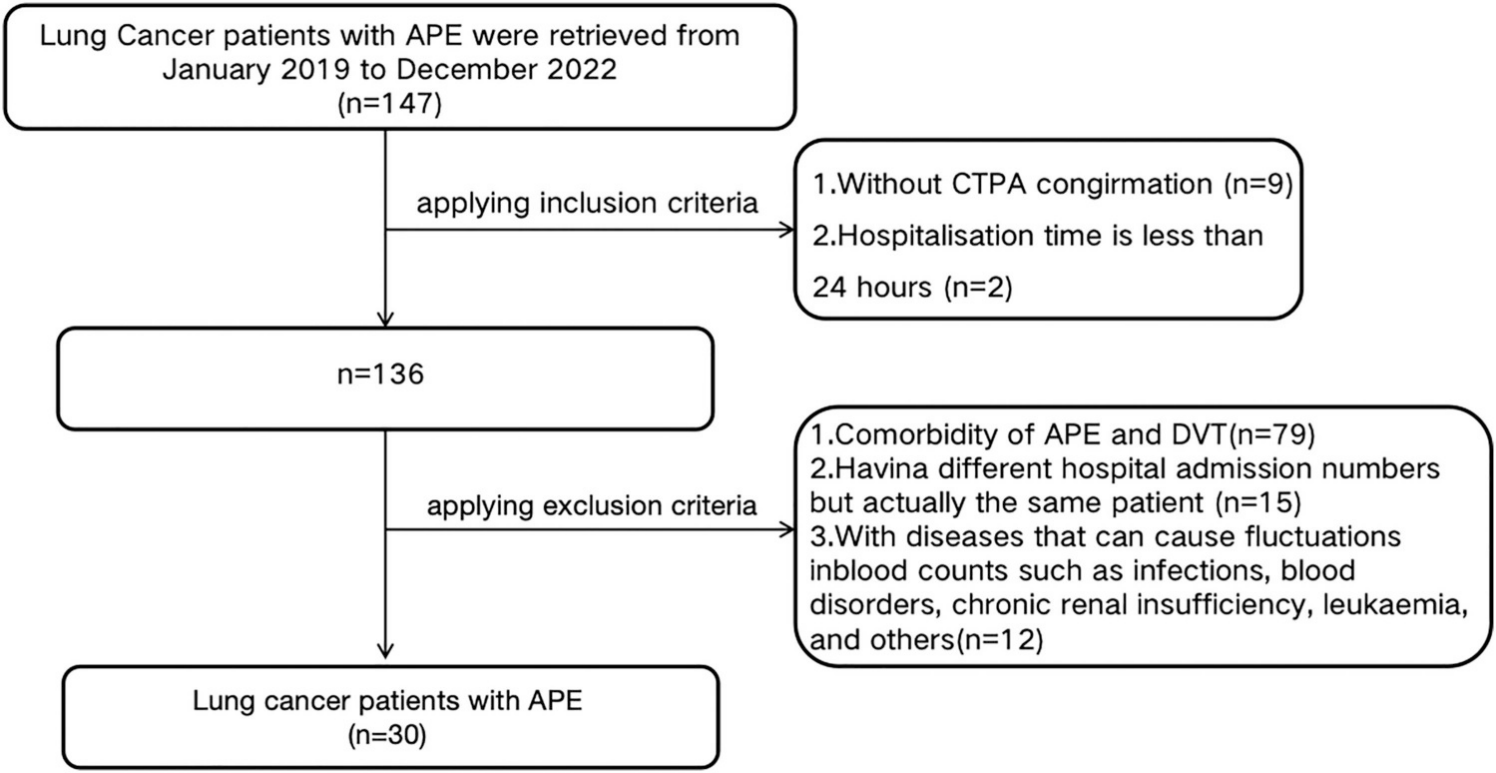
Flow chart of the study.

Exclusion criteria encompassed: 1) Comorbidity of APE and DVT at the current presentation; 2) A history of previous DVT or APE (to minimize confounding effects from prior thrombotic events and their treatments); 3) Having different hospital admission numbers, but actually the same patient; 4) combined with diseases that can cause fluctuations in blood counts such as infections, blood disorders, chronic renal insufficiency, leukaemia, and others. The flowchart was illustrated as Figure 1.

### Data collection and definition

We identified lung cancer cases from the electronic medical record system of the Second Xiangya Hospital of Central South University with ICD-10 codes. Demographic, clinical, and laboratory data were extracted, including age, gender, body mass index (BMI), medical history, vital signs, comorbidities, blood routine, coagulation function, and biochemical indices. Laboratory values, including complete blood count (CBC), coagulation parameters, and biochemical indices (including uric acid), were measured from blood samples collected at the time of hospital admission, prior to the initiation of specific therapeutic interventions for the current event (e.g., anticoagulation for APE). NLR was defined as the absolute neutrophil count divided by the absolute lymphocyte count. PLR was defined as the absolute platelet count divided by the absolute lymphocyte count.

### Statistical analysis

Characteristics were expressed as mean ± standard deviation (SD) or median (P25, P75) for continuous variables and the number of participants (percentage) for categorical variables. The statistical analysis of the differences in groups was performed by t-test or Mann–Whitney U test for continuous data and *χ*^2^ test for categorical data. The stepwise forward logistic regression model was used for identifying the risk factors of APE. The Cox proportional risk model was used for multivariate and univariate analyses to determine prognostic factors related to the disease, and hazard ratios were reported as relative risks with corresponding 95% confidence intervals. The receiver operating characteristic (ROC) curve analysis was used to determine the optimal cut-off value of index. The Kaplan–Meier method was used to estimate the proportion whether patients is admitted to the ICU unit. In this study, “poor prognosis” was primarily defined by the composite outcome of disease severity leading to ICU admission. All comparisons were employed with a two-sided test through SPSS (Statistical Package for Social Science, Chicago, IL) version 26.0, and a *P* value less than 0.05 was considered significant. All figures were concluded using the Medcalc version 20.1 software.

## Results

As shown in Figure 1, A total of 147 lung cancer patients with APE were initially identified from January 2019 to December 2022. According to the inclusion criteria, 9 patients without CTPA confirmation and 2 patients with hospitalization duration less than 24 h were excluded, leaving 136 patients. Subsequently, based on the exclusion criteria, 79 patients with comorbidity of APE and DVT, 15 patients with different hospital admission numbers but actually being the same individual, and 12 patients with diseases that could cause fluctuations in blood counts (such as infections, blood disorders, chronic renal insufficiency, leukaemia, and others) were excluded. Thirty lung cancer patients with APE (APE group) were eventually retrieved in this study after implement the inclusion and exclusion criteria. By using Propensity Score Matching (PSM), thirty lung cancer patients with DVT (DVT group) and thirty lung cancer patients without VTE (Non-VTE group) were retrieved in this study.

### Patient demographics

The demographical data of the enrolled patients are summarized in Table 1. The APE group patients were an average of 58.50 (54.75, 67.00) years old and composed of a relative even split of males (56.7%) and females (43.3%). Age, sex, BMI, vital signs, and previous history (exclude admitted to ICU, *p* < 0.001) were similar between three groups. However, the APE group exhibited a higher proportion of shortness of breath (46.7% vs. 13.3%, *p* = 0.010), chest distress (20% vs. 0, *p* = 0.024) and lower proportion of legs pain (0 vs. 13.3% and 0, *p* = 0.032) levels when compared to the Non-VTE group. When the APE group compare to DVT group, it has higher proportion of surgical treatment (46.7% vs. 13.3%, *p* = 0.005) and lower proportion of chemotherapy (13.3% vs. 40%, *p* = 0.020).

**Table 1:** Comparison of general characteristics between groups.

Variables	APE (*n* = 30)	DVT (*n* = 30)	Non-VTE (*n* = 30)	*P* value among three groups
Demographics				
Age (years)	58.50 (54.75, 67.00)	63.5 (56.00, 70.25)	59.00 (54.75, 67.00)	0.369
Sex (Male)	17 (56.7%)	20 (66.7%)	17 (56.7%)	1.000
BMI (kg/m^2^)	27.31 (25.52, 28.79)	28.04 (24.87, 29.47)	26.21 (23.47, 29.41)	0.466
Pulse	92.69 ± 15.71	93.83 ± 18.93	87.57 ± 13.51	0.332
MAP (mmHg)	99.70 ± 13.90	93.87 ± 12.34	99.11 ± 15.14	0.210
Previous history, *n* (%)				
Smoking history	14 (46.7%)	12 (40%)	14 (46.7%)	0.835
History of alcoholism	8 (26.7%)	7 (23.3%)	3 (10%)	0.233
History of operation	12 (40%)	8 (26.7%)^b^	10 (33.3%)	0.549
Emergency admission	2 (6.7%)	1 (3.3%)	0 (0%)	0.770
Clinical symptoms				
Shortness of breath	14 (46.7%)	7 (23.3%)	4 (13.3%)^c^	0.012
Hemoptysis	2 (6.7%)	2 (6.7%)	1 (3.3%)	1.000
Chest pain	4 (13.3%)	2 (6.7%)	3 (10%)	0.905
Chest distress	6 (20%)	1 (3.3%)	0 (0%)^c^	0.015
Syncope	0 (0%)	1 (3.3%)	0 (0%)	1.000
Legs edema	1 (3.3%)	1 (3.3%)	0 (0%)	1.000
Legs Pain	0 (0%)	4 (13.3%)	0 (0%)	0.032
Comorbidity, *n* (%)				
Hypertension	6 (20%)	12 (40%)	8 (26.7%)	0.220
Coronary heart disease	2 (6.7%)	3 (10%)	0 (0%)	0.363
Diabetes	1 (3.3%)	3 (10.0%)	2 (6.7%)	0.868
COPD	3 (10%)	4 (13.3%)	1 (3.3%)	0.522
Pneumonia	4 (13.3%)	3 (10%)	3 (10%)	1.000
Autoimmune disease	0 (0%)	0 (0%)	1 (3.3%)	1.000
HLP	3 (10%)	1 (3.3%)	1 (3.3%)	0.613
Therapy methods				
Surgical treatment	14 (46.7%)^a^	4 (13.3%)	10 (33.3%)	0.019
Radiotherapy	0 (0%)	2 (6.7%)	0 (0%)	0.326
Chemotherapy	4 (13.3%)^a^	12 (40%)	7 (23.3%)	0.057
Immunotherapy	2 (6.7%)	5 (16.7%)	4 (13.3%)	0.611
Targeted therapy	1 (3.3%)	5 (16.7%)	2 (6.7%)	0.263
Antiangiogenic therapy	1 (3.3%)	6 (20%)	1 (3.3%)	0.046

APE, acute pulmonary embolism; DVT, deep vein thrombosis; VTE, venous thrombus embolism; BMI, body mass index; MAP, Mean Arterial Pressure; ICU, intensive care unit; COPD, chronic obstructive pulmonary disease; HLP, hyperlipidemia.

^a^ APE group vs. DVT group;

^b^ DVT group vs. non-VTE group was statistical significance;

^c^ APE vs. non-VTE was statistical significance.

### Patient clinical indices

The clinical indices of the enrolled patients are summarized in Table 2. Statistically significant differences were observed in white blood cell (WBC, *p* = 0.028), red blood cell (RBC, *p* = 0.025), neutrophil (*N*, *p* = 0.011), NLR (*p* < 0.037), PLR (*p* < 0.001), uric acid (UA, *p* = 0.020), and estimated glomerular filtration rate (eGFR, *p* = 0.025) between the APE and DVT groups.

**Table 2:** Comparison of clinical indices between groups.

Variables	APE (*n* = 30)	DVT (*n* = 30)	Non-VTE (*n* = 30)	*P* value among three groups
Blood routine				
WBC (10^9^/L)	12.04 (9.79, 14.49)^a^	8.51 (6.32, 13.14)^b^	5.54 (4.45, 6.94)^c^	<0.001
RBC (10^12^/L)	4.01 ± 0.52^a^	3.61 ± 0.78^b^	4.39 ± 0.49^c^	<0.001
PLT (10^9^/L)	258.43 ± 112.54	235.67 ± 95.61	203.93 ± 57.8°^c^	0.074
N (10^9^/L)	10.84 (8.03, 12.36)^a^	6.91 (4.39, 10.92)^b^	3.58 (2.42, 4.29)^c^	<0.001
L (10^9^/L)	1.03 (0.69, 1.51)	1.21 (0.88, 1.71)^b^	1.51 (1.26, 1.74)^c^	<0.001
NLR	10.80 (6.89, 17.76)^a^	5.60 (3.13, 10.58)^b^	2.17 (1.52, 2.80)^c^	<0.001
E (10^9^/L)	0.30 (0.00, 0.12)	0.11 (0.03, 0.21)	0.16 (0.09, 0.24)^c^	0.001
M (10^9^/L)	0.58 ± 0.33	0.55 ± 0.20^b^	0.35 ± 0.12^c^	<0.001
PLR	229.70 (152.39, 370.00)^a^	179.35 (125.08, 260.98)^b^	130.3 (96.73, 160.10)^c^	<0.001
Coagulation function				
D-Dimer	1.80 (0.96, 40.10)	2.89 (1.16, 8.62)^b^	0.40 (0.25, 1.20)^c^	<0.001
APTT (s)	35.25 (28.98, 40.10)	34.1 (27.50, 41.45)	28.85 (27.50, 35.83)^c^	0.040
PT (s)	12.75 (10.08, 14.38)	13.05 (11.40, 15.03)^b^	11.00 (10.45, 13.20)^c^	0.009
FIB (g/L)	3.68 (2.65, 5.35)	3.87 (2.91, 5.87)^b^	2.78 (2.40, 3.70)^c^	0.003
INR	0.99 (0.92, 1.12)	1.08 (0.96, 1.18)^b^	0.96 (0.91, 1.02)	0.012
Biochemical index				
ALT	25.15 (15.70, 43.08)	18.35 (14.38, 39.28)	15.45 (11.68, 23.45)^c^	0.028
AST	28.00 (20.73, 48.18)	26.25 (17.70, 31.53)^b^	18.50 (15.95, 23.68)^c^	<0.001
AST/ALT	1.25 ± 0.46	1.28 ± 0.48	1.21 ± 0.33	0.802
ALB	34.09 ± 4.20	34.97 ± 4.82^b^	38.53 ± 3.8°^c^	<0.001
TBil (umol/l)	9.50 (6.63, 14.83)	8.30 (7.10, 10.70)	8.50 (6.70, 11.80)	0.628
DBil	3.20 (2.30, 5.68)	2.95 (2.25 4.53)	2.65 (2.20 3.75)	0.325
UA (umol/l)	231.08 ± 99.33^a^	294.48 ± 106.48	306.25 ± 70.28^c^	0.005
eGFR (ml/min/1.73 m^2^）	97.08 ± 14.19^a^	86.50 ± 20.86	92.31 ± 12.91	0.048

APE, acute pulmonary embolism; DVT, deep vein thrombosis; VTE, venous thrombus embolism; WBC, white blood cell; RBC, red blood cell; PLT, platelet; N, neutrophil; L, lymphocyte; NLR, neutrophil/lymphocyte ratio; E, eosinophil; M, monocyte; PLR, platelet/lymphocyte ratio; APTT, activated partial thromboplastin time; PT, prothrombin time; FIB, fibrinogen; INR, international standardized ratio; ALT, alanine aminotransferase; AST, aspartic transaminase; ALB, albumin; TBil, total bilirubin; UA, uric acid; eGFR, estimated glomerular filtration rate.

^a^ APE group vs. DVT group was statistical significance;

^b^ DVT group vs. non-VTE group was statistical significance;

^c^ APE vs. non-VTE was statistical significance.

Additionally, statistically significant differences were found in WBC (*p* < 0.001), RBC (*p* < 0.001), N (*p* < 0.001), L (*p* = 0.026), NLR (*p* < 0.001), monocyte (M, *p* < 0.001) count, PLR (*p* < 0.001), D-Dimer (*p* < 0.001), prothrombin time (PT, *p* = 0.003), fibrinogen (FIB, *p* < 0.001), international standardized ratio (INR, *p* = 0.003), aspartic transaminase (AST, *p* = 0.002), and albumin (ALB, *p* < 0.008) between the DVT and non-VTE groups.

Moreover, statistically significant differences were noted in WBC (*p* < 0.001), RBC (*p* = 0.005), platelet (PLT, *p* = 0.022), N, L (*p* < 0.001), NLR (*p* < 0.001), E (*p* < 0.001), M (*p* < 0.001), PLR (*p* < 0.001), D-Dimer (*p* < 0.001), activated partial thromboplastin time (APTT, *p* = 0.031), PT (*p* = 0.031), FIB (*p* = 0.016), ALT (*p* = 0.009), AST (*p* < 0.001), ALB (*p* < 0.001), and UA (*p* = 0.001) between the APE and non-VTE groups.

Clinical indices including WBC (*p* < 0.001), RBC (*p* < 0.001), N (*p* < 0.001), L (*p* < 0.001), NLR (*p* < 0.001), E, M (*p* < 0.001), PLR (*p* < 0.001), D-Dimer (*p* < 0.001), APTT (*p* = 0.040), PT (*p* = 0.009), FIB (*p* = 0.009), INR (*p* = 0.012), ALT (*p* = 0.028), AST (*p* < 0.001), ALB (*p* < 0.001), UA (*p* = 0.005), and eGFR (*p* = 0.048) showed statistically significant differences among the three groups.

WBC, RBC, N, NLR, and PLR can be found statistically significant in all of two groups comparisons and three groups comparison. CRP levels were not routinely measured in all patients at admission in this retrospective cohort and were therefore not included in the analysis.

### Independent determinants among three groups

Based on the above data and inter-group comparison results, we conducted logistic regression analysis for the three groups. In the logistic regression analysis between APE and DVT groups, the adjusted stepwise forward logistic regression model indicated that the expressed level of neutrophil [odds ratio (OR) = 0.853, 95% CI: 0.741–0.982, *p* = 0.027], and red blood cell (OR = 0.418, 95% CI: 1.178–0.979, *p* = 0.045) were independent determinants, even after adjustment for demographics, therapy methods, and laboratory indicators. In the logistic regression analysis between DVT and non-VTE groups, the adjusted stepwise forward logistic regression model indicated that the expressed level of neutrophil (OR = 1.959, 95% CI: 1.102–3.484, *p* = 0.022), and D-Dimer (OR = 1.301, 95% CI: 1.017–1.664, *p* = 0.036) were independent determinants, even after adjustment for demographics, therapy methods, and laboratory indicators. In the logistic regression analysis between APE and non-VTE groups, the adjusted stepwise forward logistic regression model indicated that the expressed level of neutrophil (OR = 3.068, 95% CI: 1.472–6.394, *p* = 0.003) and aspartic transaminase (OR = 1.268, 95% CI: 1.012–1.589, *p* = 0.039) were independent determinants, even after adjustment for demographics, therapy methods, and laboratory indicators, as shown in Table 3.

**Table 3:** Logistic regression model analysis between three groups.

Groups	Influencing factors	Adjusted model^a^	
		Odds Ratio (95% CI)	*P* value
APE vs. DVT	N	0.853 (0.741, 0.981)	0.027
	RBC	0.418 (0.178, 0.979)	0.045
DVT vs. non-VTE	N	1.959 (1.102, 3.484)	0.022
	D- Dimer	1.301 (1.017, 1.664)	0.036
APE vs. non-VTE	N	3.068 (1.472, 6.394)	0.003
	AST	1.268 (1.012, 1.589)	0.039

APE, acute pulmonary embolism; DVT, deep vein thrombosis; VTE, venous thrombus embolism; RBC, red blood cell; PLT, platelet; N, neutrophil; AST, aspartic transaminase.

^a^ Adjusted for age, sex, BMI, and statistical significant variables were compared between groups.

### Ability of neutrophil count to identify VTE condition in lung cancer patients

**Figure 2:**
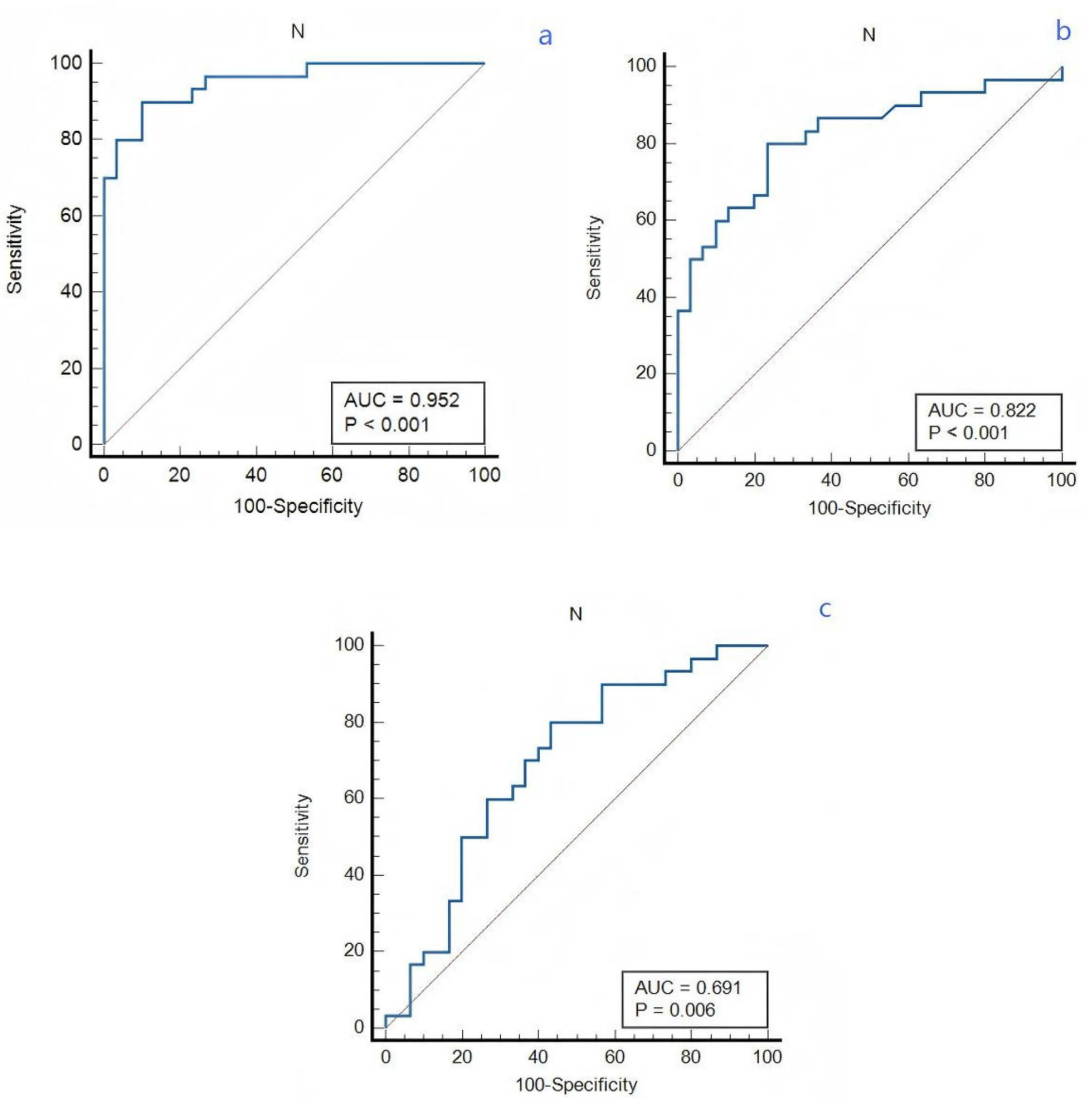
The predictive effect of neutrophil count in lung cancer patients. **(a)** APE and non-VTE groups. **(b)** DVT and non-VTE groups. **(c)** APE and DVT groups.

The receiver operating characteristic (ROC) curves was constructed for neutrophil count to predict VTE in lung cancer patients, as depicted in Figure 2. Receiver operating characteristic (ROC) curves analyzing the predictive ability of neutrophil count for differentiating between lung cancer patient groups. The area under curve (AUC) values were 0.691 (APE group vs. DVT group), 0.822 (DVT group vs. non-VTE group) and 0.952 (APE group vs. non-VTE group), respectively. The sensitivity and specificity values for APE and DVT groups are 80% and 56.7%. The sensitivity and specificity values for APE and non-VTE groups are 90% and 90%. The sensitivity and specificity values for DVT and non-VTE groups are 80% and 76.7%.

### Subgroup analysis for the APE group

According to the patients whether admitted to ICU, the APE groups was divided into the APE-ICU and the APE-nonICU groups. The demographical data and clinical indices of the enrolled patients were summarized in Tables 4, 5. A statistically significant difference was found in the history of operation (*p* = 0.007). When comparing the clinical indicators between the two groups, statistically significant differences were observed in PLT (*p* = 0.015), M (*p* = 0.023), PLR (*p* = 0.008), PT (*p* = 0.032), UA (*p* < 0.001), and eGFR (*p* = 0.002). In the multivariate analysis between those two groups (Table 6), the adjusted stepwise forward Cox proportional hazards model revealed that higher expressed levels of UA [hazard ratio (HR): 1.006, 95% confidence interval (CI): 1.000–1.012, *p* = 0.035] were associated with an increased hazard of lung cancer patients with APE which was independent from age, BMI, and sex. And it (HR: 1.006; 95% CI: 1.000–1.012, *p* = 0.035) was still shown as an independent risk factor even after adjustment for age, BMI, sex, PLR, PLT, M, PT and eGFR. Furthermore, in the multivariate analysis between APE-ICU and APE-nonICU patients, the adjusted stepwise forward logistic regression model indicated that the expressed level of UA [odds ratio (OR) = 1.017, 95% CI: 1.002–1.033, *p* = 0.028] and PLR (OR = 0.990, 95% CI: 0.981–0.999, *p* = 0.037) were associated with the severity of APE, even after adjustment for demographics, PLT, M, PT, and eGFR.

**Table 4:** Comparison of general characteristics between APE-ICU with APE-nonICU groups.

Variables	APE-ICU (*n* = 18)	APE-nonICU (*n* = 12)	*P* value
Demographics			
Age (years)	57.33 ± 10.22	62.83 ± 7.48	0.122
Male	8 (44.4%)	9 (75.0%)	0.098
BMI (kg/m2)	27.67 (25.90, 28.64)	26.39 (24.38, 28.86)	0.472
Pulse	91.90 ± 16.50	91.17 ± 17.27	0.908
MAP (mmHg)	100.76 ± 13.44	98.11 ± 15.04	0.618
Previous history, *n* (%)			
Smoking history	6 (33.3%)	8 (66.7%)	0.073
History of alcoholism	3 (16.7%)	4 (41.7%)	0.210
History of operation	11 (61.1%)	1 (8.3%)	0.007
Emergency admission	2 (22.2%)	0 (0.0%)	0.503

APE, acute pulmonary embolism; BMI, body mass index; MAP, Mean Arterial Pressure; ICU, intensive care unit.

**Table 5:** Comparison of clinical indices between APE-ICU and APE-nonICU groups.

Variables	APE-ICU (*n* = 18)	APE-nonICU (*n* = 12)	*P* value
Blood routine			
WBC (10^9^/L)	12.58 (10.75, 15.05)	11.73 (7.13, 13.86)	0.197
RBC (10^12^/L)	4.03 ± 0.47	3.97 ± 0.64	0.743
PLT (10^9^/L)	218.56 ± 92.77	318.25 ± 116.55	0.015
N (10^9^/L)	10.47 (9.10, 12.82)	10.23 (5.46, 12.05)	0.271
E (10^9^/L)	0.01 (0.00, 0.08)	0.085 (0.015, 0.23)	0.063
M (10^9^/L)	0.69 ± 0.33	0.42 ± 0.26	0.023
PLR	185.18 (145.88, 283.47)	379.88 (195.23, 493.53)	0.008
Coagulation function			
D-Dimer	1.80 (0.86, 4.61)	1.86 (1.00, 4.79)	0.672
APTT (s)	31.35 (27.88, 43.15)	38.00 (34.60, 39.38)	0.083
PT (s)	12.21 ± 1.91	13.73 ± 1.62	0.032
FIB (g/L)	2.99 (2.60, 5.25)	4.33 (3.22, 5.46)	0.271
INR	0.96 (0.91, 1.11)	1.01 (0.93, 1.17)	0.204
Biochemical index			
ALB	33.73 ± 4.28	34.83 ± 4.34	0.500
UA (umol/l)	184.18 ± 81.16	301.43 ± 82.67	<0.001
eGFR (ml/min/1.73 m^2^）	102.23 (95.60, 111.13)	92.76 (85.41, 99.91)	0.022

APE, acute pulmonary embolism; DVT, deep vein thrombosis; VTE, venous thrombus embolism; WBC, white blood cell; RBC, red blood cell; PLT, platelet; N, neutrophil; L, lymphocyte; NLR, neutrophil/lymphocyte ratio; E, eosinophil; M, monocyte; PLR, platelet/lymphocyte ratio; APTT, activated partial thromboplastin time; PT, prothrombin time; FIB, fibrinogen; INR, international standardized ratio; TBil, total bilirubin; UA, uric acid; eGFR, estimated glomerular filtration rate.

**Table 6:** Multiple regression on model analysis between APE-ICU and APE-nonICU groups.

Adjusted model^a^			
Multiple regression	Influencing factors	Hazard ratio or odds ratio (95% CI)	*P* value
Cox proportional hazards model	UA	1.006 (1.000, 1.012)	0.035
Logistic regression model	PLR	0.990 (0.981, 0.999)	0.037
	UA	1.017 (1.002, 1.033)	0.028

APE, acute pulmonary embolism; ICU, intensive care unit; PLR, monocyte/lymphocyte ratio; UA, uric acid.

^a^ Adjusted for age, sex, BMI, and statistical significant variables were compared between groups.

### Ability of UA and PLR to identify the severity for lung cancer patients with APE

**Figure 3:**
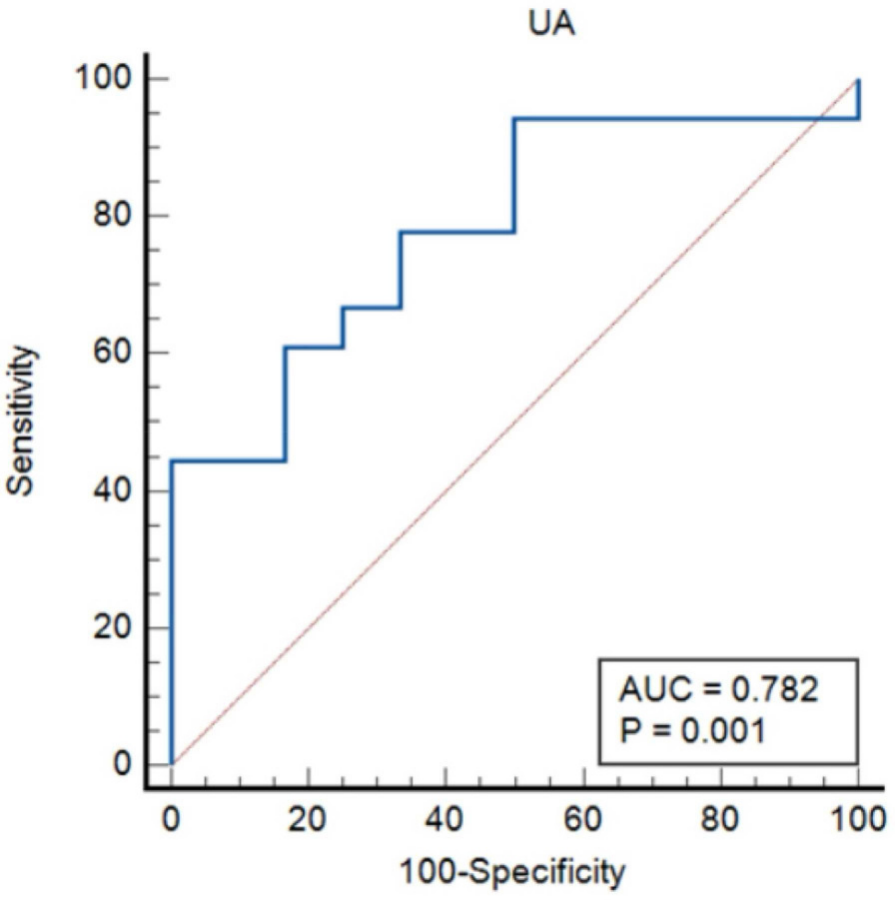
The predictive effect of UA in lung cancer patients with APE.

**Figure 4:**
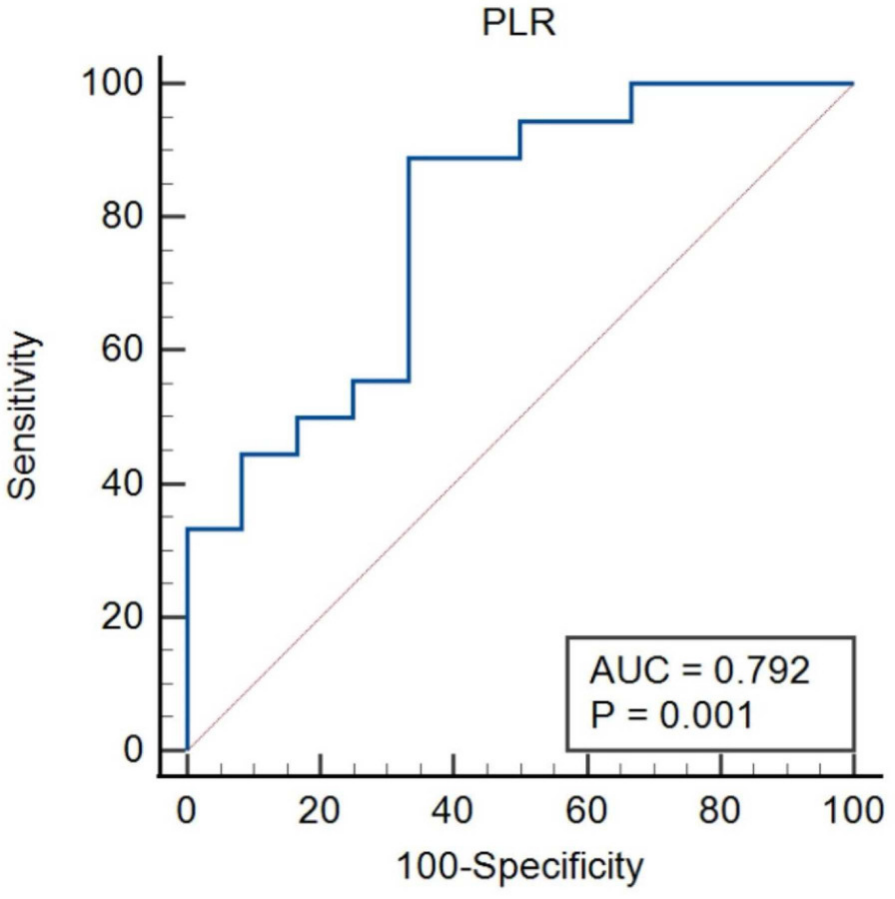
The predictive effect of PLR in lung cancer patients with APE. PLR, platelet/lymphocyte ratio.

The ROC curves were constructed for UA and PLR to predict severity in lung cancer patients with APE, as depicted in Figuress 3, 4. In Figure 3, ROC curve for Uric Acid (UA) predicting ICU admission (severity) in lung cancer patients with APE (AUC = 0.782, *p* = 0.001). In Figure 4, ROC curve for Platelet-to-Lymphocyte Ratio (PLR) predicting ICU admission (severity) in lung cancer patients with APE (AUC = 0.792, *p* = 0.001). The end-off time is defined as the duration from the time of VTE diagnosis to ICU admission. If the patient was diagnosed after entering the ICU, the end-off time was considered as the 0 day. The AUC value for UA was 0.782 and *P* value was 0.001. The AUC value for PLR was 0.792 and *P* value was 0.001. Meanwhile, the optimal predictive threshold for the expressed level of UA was determined to be 260.1 µmol/L according to the AUC, yielding a sensitivity of 61.11% and specificity of 83.33%. The optimal predictive threshold for the expressed level of PLR was determined to be 318.83 according to the AUC, yielding a sensitivity of 88.89% and specificity of 66.67%.

### The severity of APE assessment based on the levels of PLR and UA in different groups

**Figure 5:**
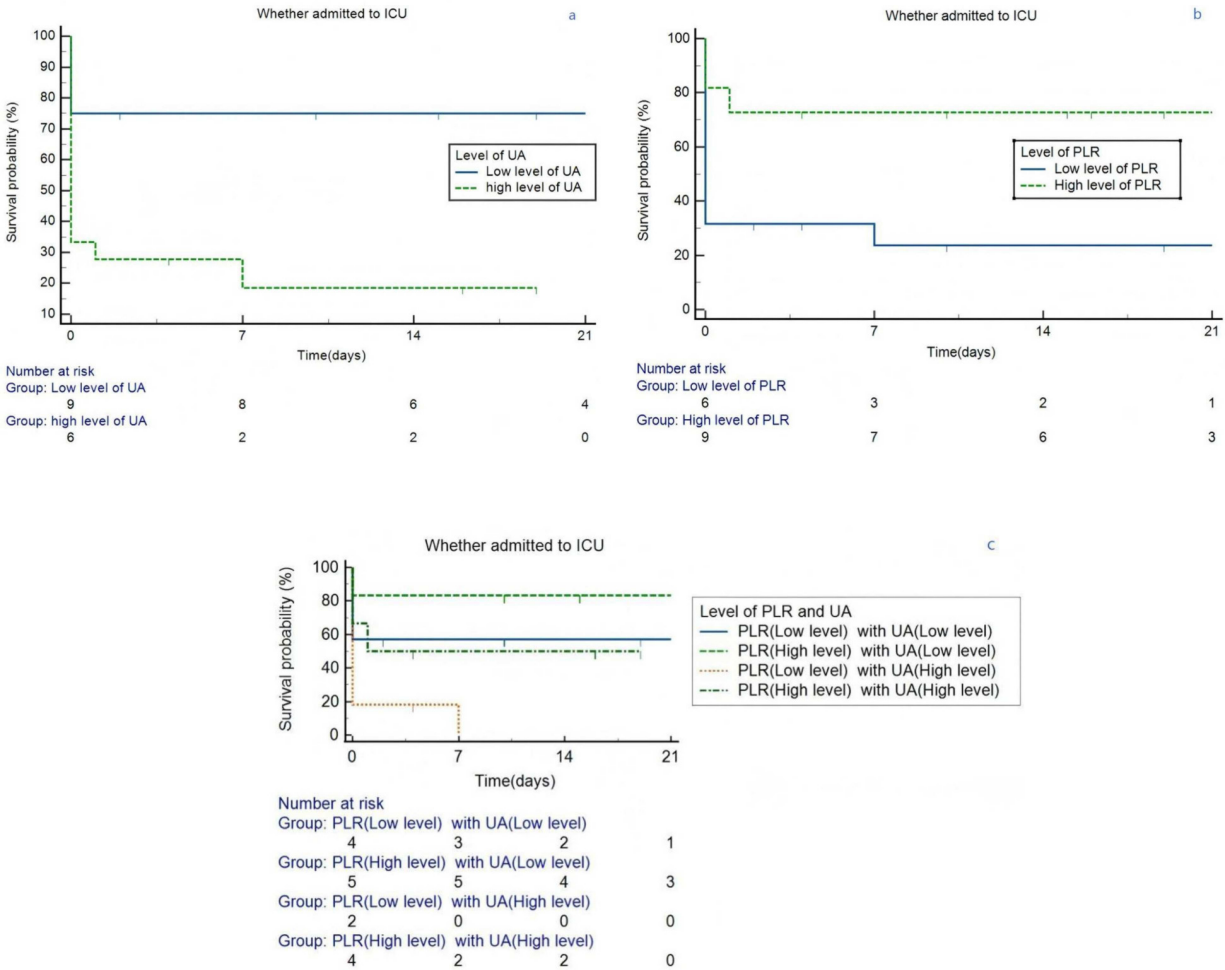
Kaplan–Meier survival curves in lung cancer patients grouped by the level of PLR and UA. **(a)** Different level of UA. **(b)** Different level of PLR. **(c)** Different level of PLR and UA. ICU, intensive care unit; PLR, platelet/lymphocyte ratio; UA, uric acid.

Kaplan–Meier analysis was conducted base on different level of PLR (*p* = 0.005) and UA (*p* = 0.004), as separately depicted in Figures 5a, b. Kaplan–Meier curves for ICU admission-free survival in lung cancer patients with APE, stratified by (a) Platelet-to-Lymphocyte Ratio (PLR) levels (cut-off 318.83, Log-rank *p* = 0.005), (b) Uric Acid (UA) levels (cut-off 260.1 µmol/L, Log-rank *p* = 0.004), and (c) the combination of high UA and low PLR (Log-rank *p* = 0.009). The graphs are of uniform size, and labels (a, b, c) are positioned above the respective panels. Subsequently, the lung cancer patients with APE of UA (associated criterion > 260.1 µmol/L) were compared to those with PLR (associated criterion ≤ 381.83). The difference in ICU admitted proportion was statistically significant (*p* = 0.009, Figure 5c).

## Discussion

This study highlights the interplay between inflammation and thrombosis in lung cancer patients with APE. Inflammation is a fundamental response of the immune system to injury or infection, playing a crucial role in various disease processes (21). There is substantial evidence that inflammatory environments themselves can be prothrombotic, acting as an intravascular effector of innate immunity (22). It has been known for quite some time that such a bidirectional link exists between inflammation and VTE. A number of studies suggest that a thrombotic event induce an inflammatory status that favours the development of post-thrombotic syndrome (23). The change in inflammatory factors provides valuable insights into the underlying inflammatory processes associated with thrombosis and can aid in the diagnosis and management of patients with VTE (24).

Inflammation mediators and cellular effectors are crucial in the tumour microenvironment (25). In the context of cancer, inflammation has been shown to contribute to tumour progression and metastasis (26). In certain types of cancer, inflammatory conditions precede malignant transformation (27). Conversely, in other types of cancer, oncogenic changes induce an inflammatory microenvironment that facilitates tumour development (28). It contributes to the proliferation and survival of malignant cells, promotes angiogenesis and metastasis, suppresses adaptive immune responses, and modifies responses to hormones and chemotherapeutic agents. APE characterized by the obstruction of blood vessels in the lungs, can further exacerbate the already dire situation for lung cancer patients (13, 29). Although previous research did not find a significant correlation between inherited thrombophilic mutations and ESC 2019 PE risk categories, our findings suggest a need for further exploration of how genetic risk factors may contribute to individualized risk assessment (30).

Neutrophils play a crucial role in the body's immune response and inflammation. Neutrophil infiltration into the tumour has been consistently associated with poorer patient outcomes (31). Recent study found that higher neutrophil counts are associated with atherosclerotic cardiovascular disease, implying that a high neutrophil count is a causal risk factor for atherosclerotic cardiovascular disease (32). There are few studies suggested that neutrophils also contribute to the development of VTE. During VTE, neutrophils become activated and recruited to the site of thrombus formation. Neutrophils can also interact with platelets and endothelial cells, further enhancing the pro-thrombotic environment (23, 33). Furthermore, neutrophils release neutrophil extracellular traps (NETs), which are web-like structures composed of DNA, histones, and granular proteins. Previous study reveals that NETs might play a role in cancer-related coagulopathy (34). These studies mechanistically explain the findings in our study.

UA, the final product of purine metabolism, has been demonstrated to influence the interaction among compromised endothelial function, inflammatory response, and thrombogenicity (35). Uric acid levels can be higher in patients with higher rates of cell turnover, such as those with advanced or metastatic cancer or those undergoing certain anti-cancer treatments. While our study lacked complete detailed staging and treatment data for all patients, this potential relationship should be considered when interpreting UA levels. Numerous observational and experimental studies have suggested that hyperuricemia is associated with an increased risk of VTE (35, 36). However, these associations may be influenced by various confounding factors that are challenging to fully consider in these studies, and the possibility of reverse causality cannot be eliminated. Most clinical observational studies support a positive correlation between UA and VTE risk, which aligns with our findings (37–40). Our results confirm that this conclusion is also applicable in lung cancer patients with VTE.

PLR, which integrates the detrimental effects of neutrophilia or thrombocytosis and lymphopenia, have emerged as potentially useful prognostic parameters in cancer patients (41, 42). A cohort study was conducted to retrospectively analyze NLR and PLR in 810 consecutive cancer out-patients with primary or relapsing solid cancer at the start of a new chemotherapy regimen, hinted that PLR might represent a yet unrecognized risk factor for VTE in cancer out-patients receiving chemotherapy (43). Our study fulfills the gap in this area, suggesting that low levels of PLR may increase the severity and poor prognosis of APE.

## Conclusion

This study aims to investigate the impact of inflammatory factors on lung cancer patients with pulmonary embolism. By analyzing the underlying association between clinical laboratory indices and disease progression, we sought to provide valuable insights for the prophylaxis and treatment for of this population. The ultimate objective is to establish reliable screening methods and predictive models that can support clinicians in formulating individualized treatment and management plans As one of the few studies focusing on the role of inflammation in lung cancer patients with APE, our findings reveal a significant association between elevated neutrophil counts and the occurrence of APE, suggesting its potential role as a risk indicator. Additionally, low PLR and high UA levels may reflect greater disease severity and poorer prognosis in APE patients.

### Limitation

This study has several limitations. First, the sample size was relatively small, which was attributable to the strict inclusion criteria requiring definitive diagnosis, detailed clinical examination results, and minimization of potential confounding factors in this single-center study. Furthermore, important variables such as detailed cancer stage, specific cancer treatment regimens, and the presence of concurrent DVT at diagnosis could not be fully accounted for in the analysis due to missing data or sample size constraints, which may limit the validity and generalizability of the findings. The lack of data on additional inflammatory markers like CRP is another limitation. In addition, the lack of long-term follow-up limited our ability to evaluate prognostic outcomes over time. Future prospective studies should prioritize larger sample sizes, standardized data collection on cancer characteristics, treatment details, and inflammatory biomarkers, as well as incorporate extended follow-up to improve the generalizability and clinical relevance of the findings.

## Data Availability

The data that support the findings of this study are available from the corresponding author upon reasonable request due to ethical restrictions.
